# Large language model doctor: assessing the ability of ChatGPT-4 to deliver interventional radiology procedural information to patients during the consent process

**DOI:** 10.1186/s42155-024-00477-z

**Published:** 2024-11-29

**Authors:** Hayden L. Hofmann, Jenanan Vairavamurthy

**Affiliations:** 1 Keck School of Medicine, University of Southern California, Los Angeles, CA USA; 2 Icahn School of Medicine at Mount Sinai, New York, NY USA

**Keywords:** ChatGPT4, Large Language Model, Informed Consent, Interventional, Radiology, Artificial Intelligence

## Abstract

**Purpose:**

The study aims to evaluate how current interventional radiologists view ChatGPT in the context of informed consent for interventional radiology (IR) procedures.

**Methods:**

ChatGPT-4 was instructed to outline the risks, benefits, and alternatives for IR procedures. The outputs were reviewed by IR physicians to assess if outputs were 1) accurate, 2) comprehensive, 3) easy to understand, 4) written in a conversational tone, and 5) if they were comfortable providing the output to the patient. For each criterion, outputs were measured on a 5-point scale. Mean scores and percentage of physicians rating output as sufficient (4 or 5 on 5-point scale) were measured. A linear regression correlated mean rating with number of years in practice. Intraclass correlation coefficient (ICC) measured agreement among physicians.

**Results:**

The mean rating of the ChatGPT responses was 4.29, 3.85, 4.15, 4.24, 3.82 for accuracy, comprehensiveness, readability, conversational tone, and physician comfort level, respectively. Percentage of physicians rating outputs as sufficient was 84%, 71%, 85%, 85%, and 67% for accuracy, comprehensiveness, readability, conversational tone, and physician comfort level, respectively. There was an inverse relationship between years in training and output score (coeff = -0.03413, *p* = 0.0128); ICC measured 0.39 (*p* = 0.003).

**Conclusions:**

GPT-4 produced outputs that were accurate, understandable, and in a conversational tone. However, GPT-4 had a decreased capacity to produce a comprehensive output leading some physicians to be uncomfortable providing the output to patients. Practicing IRs should be aware of these limitations when counseling patients as ChatGPT-4 continues to develop into a clinically usable AI tool.

## Introduction

The chatbot nature of popular open access large language models (LLMs) such as Chat Generative Pre-Trained Transformer (ChatGPT) has garnered much attention for its ability to produce accurate and coherent information for a wide variety of medical inquiries. As these models rapidly evolve, researchers are eager to understand how to harness the power of these models while also investigating potential weaknesses. In March of 2023, OpenAI released its most recent LLM entitled Chat Generative Pre-Trained Transformed 4 (ChatGPT-4). The updated model was trained on an extensive dataset and demonstrated an improved ability to understand and generate text for more complex scenarios [1]. The latest model has received widespread recognition given its greatly improved text comprehension and production to create more accurate outputs. ChatGPT-4’s increased output accuracy has led to higher performance on the United States Medical Licensing Examination (USMLE) and on radiology board exams increasing its score from 69% with ChatGPT-3 to 81% with ChatGPT-4 [2, 3].

Previous studies have analyzed ChatGPT for potential application in patient education. Specifically in the field of interventional radiology (IR), ChatGPT was found to provide reliable information regarding IR-related content, however, in some instances, the information can be both inaccurate and confusing [4, 5]. Other studies have highlighted the potentially dangerous shortcomings of ChatGPT, such as its generation of nonexistent references for medical diagnoses and treatments [6]. Given the free and open-access nature of the model, patients may utilize this tool to research their conditions making them susceptible to misinformation regarding their conditions and treatment options. According to recent CDC reports, over 58% of US adults have used the Internet to find medical information [7]. As LLMs continue to grow in popularity, more patients may look to ChatGPT’s interactive interface to find answers to their medical questions.

The updates to the ChatGPT model as well as the increasing popularity and widespread knowledge of this new LLM technology provide an opportunity to reassess how ChatGPT can be further used in informed consent and patient education within interventional radiology. ChatGPT’s accurate and human-like outputs through a chatbot user interface could be leveraged to assist in consenting patients for certain procedures to help streamline care. Conversely, much like other medical information providers of the past, patients may get information about upcoming procedures from these LLMs, so practicing physicians should be aware of the information patients may be receiving. The purpose of this study is to evaluate how current interventional radiologists view ChatGPT outputs in the context of providing information to augment the process of informed consent for common interventional radiology procedures.

## Methods

Institutional review board (IRB) exemption was obtained. ChatGPT-4 (GPT4) was prompted to outline the risks, benefits, and alternatives for five common procedures performed by interventional radiologists: CT-guided lung biopsy, percutaneous nephrostomy tube placement, transarterial chemoembolization (TACE), inferior vena cava (IVC) filter placement, and IVC filter retrieval. The following prompt was queried: “Hello ChatGPT, you are an interventional radiologist consenting a patient for a procedure. Please write a short paragraph that outlines the risks, benefits, and alternatives of a [IR procedure] that can be given to the patient.” The following prompt were designed to first establish a role for ChatGPT to follow (you are an interventional radiologist consenting a patient) and then provide a clear objective (outline the risks, benefits, and alternatives) for the model to follow: both established methods to improve LLM output quality [8, 9]. ChatGPT outputs were reviewed by a physician which confirmed there were no hallucinations or factually incorrect or unrelated statements. The survey was distributed to a large number of interventional radiology physicians in academic settings through email and social media. No incentive was offered for completing the survey. All outputs were assessed for 1) correctness, 2) comprehensiveness, 3) readability for a standard patient, 4) physician comfort with providing the output to the patient, and 5) conversational tone. Physicians evaluated each component on a Likert Scale numbered 1 through 5 which corresponded to strongly disagree, disagree, neither agree nor disagree, agree, and strongly agree, respectively.

The percentage of graders who deemed the output sufficient, recording a 4 (agree) or 5 (strongly agree), was measured and component scores were averaged across cohorts. Interrater reliability was measured using the two-way random-effects model to calculate the intraclass correlation coefficient (ICC) using the IRR package in R [10]. Scores given by attendings and resident cohorts were compared using t-tests. Additionally, linear regression was used to compare reported years of practice and average output scores. GPT4 outputs were further analyzed for readability using common readability measurement scales such as Flesch Kincaid Grade Level, Flesch Kincaid Reading Ease, Coleman Liau Index, Gunning Fog Score, and Smog Index. All readability measurements excluding Flesch Kincaid Reading Ease are designed to approximate U.S. grade level needed to read the text. Measurements are scaled from zero being prekindergarten to eighteen being postgraduate level. Flesch Kincaid Reading Ease, on the other hand, is scaled from 1 being the most difficult to read to 100 being the easiest to read. Sentence and word count were also recorded.

## Results

**Fig. 1:**
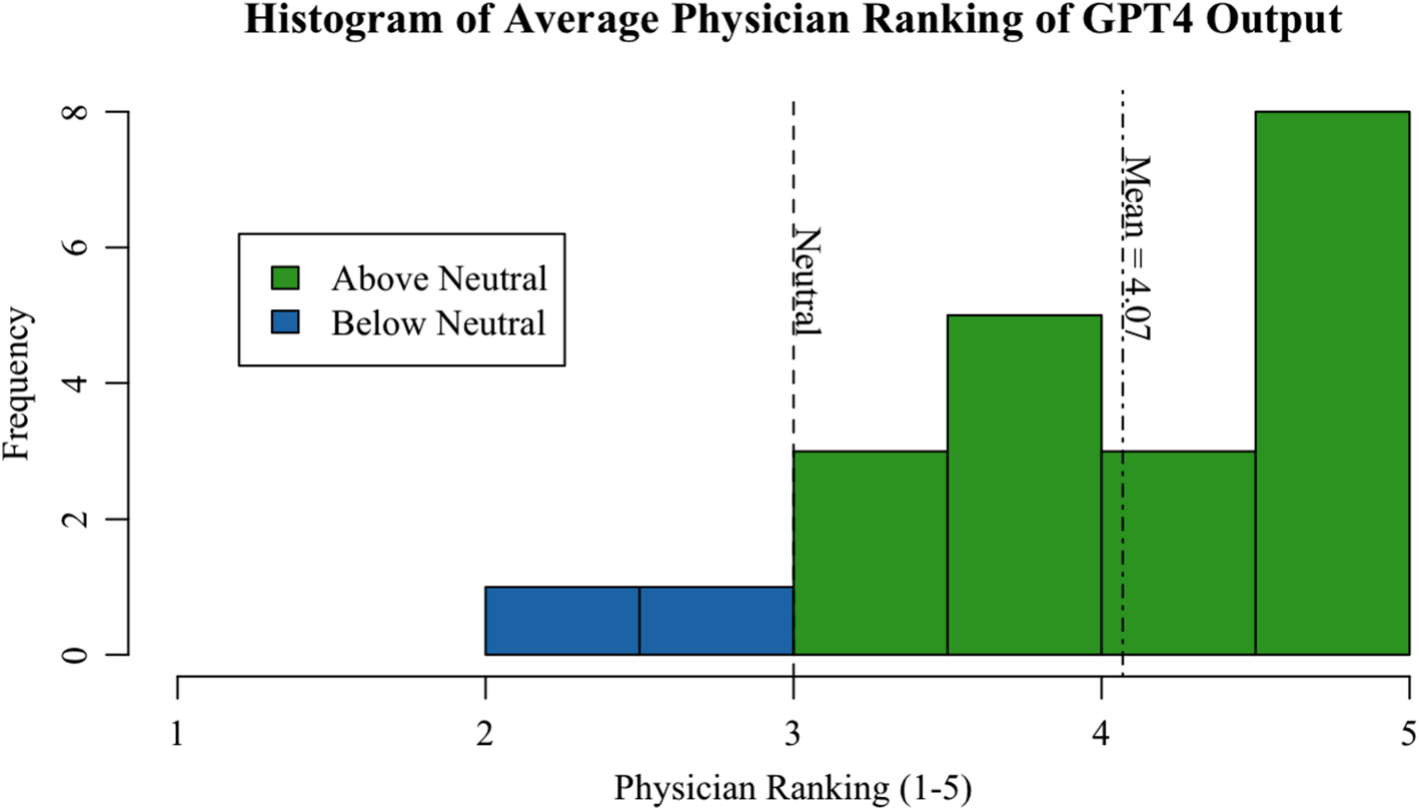
Histogram of mean physician rankings of ChatGPT-4 outputs across all five procedures. The vertical line at 3.0 represents the neutral ranking

We obtained responses from 21 physicians (*n* = 21) from six academic institutions (Keck School of Medicine of USC, Icahn School of Medicine at Mount Sinai, University of California Los Angeles, Stanford University, University of Colorado, and University of Chicago). Seven residents (33.3%) and fourteen attendings (66.7%) responded of which sixteen were male (76.2%) and five were female (23.8%). The average year in practice for physicians was 16.4 years. On a scale of 1–5, the average grade of the ChatGPT responses was 4.29 on accuracy, 3.85 on comprehensiveness, 4.15 on readability, 4.24 on conversational tone, and 3.82 on physician comfort level. Figure 1 portrays the physicians’ average ranking across all five procedures and all five evaluation metrics as a histogram with a cumulative mean (4.07) and neutral ranking (3.0). The percentage of graders who deemed the output sufficient rating a 4 (agree) or 5 (strongly agree) was 84%, 71%, 85%, 85%, and 67% for accuracy, comprehensiveness, readability, conversational tone, and physician comfort level, respectively. Further breakdown of ChatGPT scores by survey criteria is outlined in Table 1.

**Table 1:** GPT-4’s performance across various evaluation metrics

	Cumulative Mean	Lung Biopsy Mean	Nephro Tube Mean	IVC Placement Mean	IVC Retrieval Mean	TACE Mean	Attending Mean	Resident Mean	% graders $\ge$ 4 (all procedures)
Accuracy	4.29	4.52	4.33	4.24	4.05	4.29	4.09	4.69	84
Comprehensiveness	3.85	3.95	4.05	3.71	3.62	3.91	3.76	4.03	71
Readability	4.15	4.33	4.14	4.14	3.95	4.19	4.2	4.06	85
Conversational Tone	4.24	4.38	4.29	4.10	4.19	4.24	4.23	4.26	85
Comfortable providing to patient	3.82	4.10	3.95	3.62	3.62	3.81	3.73	4.00	67

**Fig. 2:**
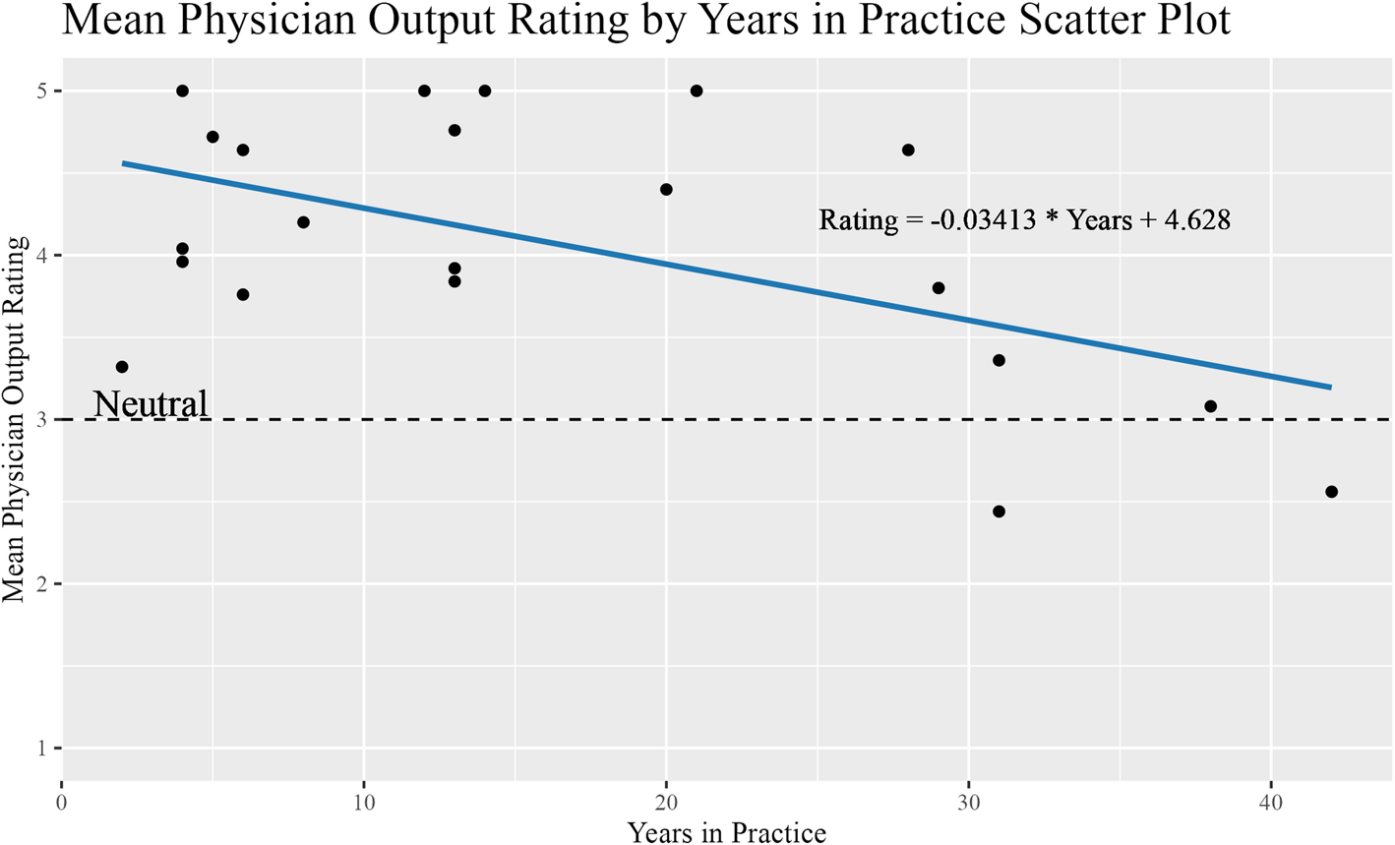
Linear Regression correlating mean physician ranking across all five procedures to number of years in practice. The horizontal line at 3.0 represents the neutral ranking

There was an inverse relationship between years in training and mean output score (coeff = -0.03413, *p* = 0.0128); Fig. 2. However, no significant difference was seen between mean output scores between attending (4.00) and resident (4.21) cohorts (*p* = 0.5367). The intraclass correlation coefficient (ICC) was 0.39 (*p* = 0.003) with 95% confidence of [0.111, 0.646] from the 25 survey question responses from each of the 21 physicians.

The mean Flesch-Kincaid grade level for the five GPT4 generated consent information was 11.65. The Flesch-Kincaid Reading Ease, Coleman Liau Index, Gunning Fog Score, and Smog Index were 42.2, 13.11, 15.38, and 11.2, respectively. The readability measures of the original ChatGPT outputs for the IR procedures can be seen in Table 2.

**Table 2:** GPT-4’s reading level performance by procedure type

	Lung Biopsy	Nephro Tube	IVC Placement	IVC Retrieval	TACE	Mean
Flesch Grade Level	12.27	10.47	11.57	11.29	12.63	11.65
Flesch Reading Ease	41	47	43	47	33	42.2
Coleman Liau Index	13.04	12.64	13.36	11.99	14.50	13.11
Gunning Fog Score	16.8	14.6	14.8	14.5	16.2	15.4
Smog Index	12.21	10.44	10.80	11.00	11.57	11.20
Word Count	243	259	255	278	255	258
Sentence Count	12	14	12	12	11	12

## Discussion

As evaluated by practicing IR physicians, ChatGPT generated information for patient consent that was accurate, written in a conversational tone, and could be understood by a standard patient. The majority of physicians agreed the outputs were accurate, conversational, and could be read by a normal patient, all critical aspects of the informed consent process. Prior studies have evaluated both ChatGPT-3 and ChatGPT-4 utility in patient consent, finding that both models accurately answered patient inquiries regarding IR procedures [11]. However, the Flesch-Kincaid Grade Level (11.65) for GPT4 was well above the recommended 8th-grade level which is in concordance with findings seen in previous studies that examine the readability of the prior ChatGPT-3 model [4]. The similarities seen in both models highlight how updates to the ChatGPT model do not necessitate improvements in all domains. Due to the shifting scopes of LLMs like ChatGPT, there is need for ongoing reassessment to evaluate the potential strengths and pitfalls of this clinically applicable tool.

Furthermore, a periodical assessment of how physicians view this new-age tool is critical as the LLMs evolve and both the capabilities and opinions on this technology change. The surveyed physicians highlighted a potential pitfall of ChatGPT-4 as a viable clinical tool: a decreased ability to provide a comprehensive explanation for the IR procedure. Nearly one-third of physicians reported that the GPT-4 output did not comprehensively explain the procedures’ risks, benefits, and alternatives. While ChatGPT-4’s outputs were rated as accurate, a significant portion of physicians reported that the information was insufficient in explaining the necessary information for a patient's consent, a finding similarly observed in the previous iteration of ChatGPT and ChatGPT-4 [5, 12]. However, this finding is not unique to ChatGPT as previous papers have discovered complications in real-life IR cases that were not initially documented during the consenting process [13, 14]. This limitation may help explain why one-third of all surveyed physicians (33%) reported that they were not comfortable providing the outputs to their patients. The data highlights the need for physician supervision and verifications of medical outputs from the current version of the model.

While physician verification of ChatGPT outputs may seem like the optimal path forward, lack of agreement across physicians may limit this implementation. The intraclass correlation coefficient (0.39) measured physician ratings across all five procedures and demonstrated poor interrater agreement. The surveying physicians were unable to agree on their evaluation of ChatGPT outputs, highlighting the subjective nature in which medical ChatGPT outputs are viewed by physicians. Similarly, poor interrater agreement has been observed in the assessment of physician responses to questions posed by patients. In an analysis of GPT-4’s ability to respond to patient messages, researchers showed that GPT-4 responses had greater interrater agreement as compared to physician generated responses [15]. The poor interrater reliability exemplifies an obstacle that, while not unique to LLM technology, must be addressed in the implementation and future deployment of LLM technology.

In analyzing the physician demographics, the linear regression revealed an inverse relationship between years in training and average output score. The fewer years in practice of the physician the higher they rated the ChatGPT output, and conversely, the more years in practice of the physician the lower they rated the output. The relationship has been observed in other studies that surveyed the general public finding that people under 50 years old are more likely to find ChatGPT highly useful than those over 50 years old [16]. With technology becoming a more integral part of medicine, it is important to convey the current state of LLM technology to the IR community. IR is a specialty built by innovation. From its beginning, IR has been a field of rapid adaptors who continuously iterate and develop more advanced ways to impact patient care by constantly pushing the envelope on what is possible. As a specialty that resides at the forefront of innovation and at the intersection of medicine and technology, IR is a field ripe to capitalize on this rapidly evolving technology.

This study was limited in its design in that physicians surveyed in the study were not blinded to the author of the outputs (ChatGPT). Therefore, raters may have been biased both positively and negatively by their preconceived views of ChatGPT and artificial intelligence. Furthermore, the twenty-one physicians all from academic institutions are unlikely to be a completely accurate representation of all practicing interventional radiologists. A larger study is needed to better capture a more representative assessment of the model through the lens of a general IR physician. Still, the present study provides a strong evaluation of the state of ChatGPT-4 that is needed to further assess how ChatGPT evolves. Furthermore, our input prompt to ChatGPT was not validated to be comprehensive, so patients may use other wording when interfacing with LLMs that may produce information not captured by our query. Additionally, the present study was not designed to capture the full complex nature of the consenting process and instead focuses on the delivery of accurate information that can be easily understood by patients. Future studies should look to assess ChatGPT-4’s capacity to engage in a reciprocal conversation as well as assess a patient’s competency to give consent.

## Conclusion

ChatGPT4 generated information for patient consent for IR procedures that the majority of physicians viewed as accurate, written in a conversational tone, and could be understood by a standard patient. However, outputs were insufficient in explaining the necessary information for a patient's consent leading IR physicians to feel uncomfortable providing the outputs to patients. Practicing interventional radiologists must acknowledge that ChatGPT-4 will be a tool patients use to garner information about procedures, and we must expose ourselves to the information they will receive. More importantly, we must continuously evaluate its thoroughness and accuracy in portraying IR procedures to adequately counsel patients. The outputs provided by LLMs are not completely comprehensive, and at times inaccurate, which may lead to a disconnect between patients and physicians in their understanding of their conditions and treatment options. IR physicians should understand both the immense promise this technology possesses in addition to its potentially damaging drawbacks.

## Data Availability

The datasets generated and/or analysed during the current study are publicly available and may be accessed in the supplemental materials.
